# Burden of subclinical coronary atherosclerosis among asymptomatic adults: the REACH–Rural India Study

**DOI:** 10.1016/j.lansea.2026.100726

**Published:** 2026-02-11

**Authors:** Avinash Kumar Raghupathy, Mohanraj Sundaresan, Pudhiavan Arun, Dharam J. Kumbhani, Manoj Kumar Baskar, Haritha Thambi, Parthasarathy Ayothi, Karthika Durairaj, Samrat Ashok Vasudevan, Srinidhi Narayani Seenivasan, Buvaneswari Gajendran, Gowdham Manivel, Gowri Subramaniam, Madhavi Sambandam, Velmurugan Ganesan, Balakumaran Jeyakumaran, Jeevithan Shanmugam, Krishnan Swaminathan, Arulraj Ramakrishnan, Mathew Cherian, Thomas Alexander

**Affiliations:** a KMCH Research Foundation, Coimbatore, India; b Department of Radiology, Kovai Medical Center and Hospital, Coimbatore, India; c UT Southwestern Medical Center, USA; d Department of Biochemistry, Dr. N.G.P. Arts and Science College, Coimbatore, India; e KMCH College of Nursing, Coimbatore, India; f Department of Cardiology, Kovai Medical Center and Hospital, Coimbatore, India; g Department of Community Medicine, KMCH Institute of Health Sciences and Research, Coimbatore, India; h Liver Unit, Kovai Medical Center and Hospital, Coimbatore, India; i Central Research Laboratory, KMCH Institute of Health Science and Research, Coimbatore, India; j KMCH College of Pharmacy, Coimbatore, India

**Keywords:** Coronary artery calcification, Rural-population, Epidemiology, Coronary artery disease

## Abstract

**Background:**

Coronary artery calcification (CAC) serves as a robust marker of subclinical atherosclerosis and an independent predictor of cardiovascular disease (CVD). Despite its clinical significance, population-based evidence on CAC prevalence and its determinants in rural India remains limited. This study aimed to estimate the prevalence of CAC and evaluate its association with cardiometabolic risk factors among adults aged 35–65 years in a rural Indian population.

**Methods:**

A total of 3006 individuals aged 35–65 years were selected and invited to participate in the REACH (Risk Evaluation of Subclinical Coronary Health)–Rural India Study. From 2022 to 2024, the study enrolled asymptomatic adults with no history of cardiovascular events from rural communities in the Coimbatore and Tirupur districts of Tamil Nadu, India. Data collection included sociodemographic and clinical profiling, laboratory testing, and carotid intima-media thickness (cIMT). CAC was quantified using the Agatston scoring method from dual-source CT scans.

**Findings:**

In total 2961 participants included in the final analysis (mean age 49.8 ± 8.3 years; 52.8% women), the overall prevalence of coronary artery calcification (CAC) was 25.6%. Prevalence was significantly higher in men (33.5%) than in women (18.5%) (p < 0.001). The prevalence of severe CAC (Agatston score >400) was threefold higher in men and most common in those aged 56–65 years. However, no detectable CAC was observed among women aged 35–45 years. The distribution of CAC scores was as follows: 7.0% had minimal CAC (1–10), 11.4% mild (11–100), 5.2% moderate (101–400), and 1.9% severe (>400). Multivariable logistic regression showed that diabetes, hypertension, overweight/obesity, and abnormal CIMT were independently associated with CAC. Among men, current smoking was also significantly associated (OR = 1.51; 95% CI: 1.18–1.93). In total, 83.5% of individuals with CAC had one or more cardiovascular risk factors. No significant associations were observed between CAC and elevated creatinine, reduced eGFR and peripheral artery disease (PAD) after full adjustment.

**Interpretation:**

This study reveals a substantial burden of CAC in a rural Indian population, with prevalence patterns comparable to urban cohorts in Western countries. The findings underscore the need to incorporate CAC screening in individuals with metabolic risk factors, especially in underserved populations, to identify early subclinical atherosclerosis and reduce CVD risk.

**Funding:**

10.13039/501100001411Indian Council of Medical Research (ICMR), (REF No. 5/4/1-9/2020-NCD-1).

**Research in context**

**Evidence before this study**

Coronary artery calcification (CAC) is a well-established marker of subclinical atherosclerosis and a powerful predictor of cardiovascular events in asymptomatic individuals. Most large population-based CAC studies, including MESA (Multi-Ethnic Study of Atherosclerosis), SCAPIS (Swedish CArdioPulmonary bioImage Study), and BioImage, have been conducted in urban, high-income, and ethnically diverse Western populations. South Asians, particularly those residing in the U.S., have shown higher CAC burden and progression rates compared to other ethnic groups. However, no prior large-scale study has evaluated the prevalence and associated risk factors of CAC in rural Indian populations, which remain underrepresented in global cardiovascular imaging research. Given the rising burden of cardiometabolic diseases in rural India, a need exists to assess subclinical coronary atherosclerosis in this setting.

**Added value of this study**

This population-based study assessed the burden of CAC in a rural Indian cohort comprising 3006 asymptomatic adults aged 35–65 years from Tamil Nadu. The study found a CAC prevalence of 25.6%, with a significantly higher burden in men (33.5%) compared to women (18.5%), and an increase with age. Notably, the prevalence and severity of CAC in this rural Indian cohort were comparable to those reported in urban and high-income countries, despite differences in socioeconomic background, access to healthcare, and lifestyle patterns. The study also provides age- and sex-specific CAC percentiles, offering valuable baseline data for future risk stratification. Furthermore, it identified associations between CAC and conventional cardiometabolic risk factors such as hypertension, diabetes, dyslipidemia, overweight/obesity, and abnormal CIMT.

**Implications of all the available evidence**

The findings emphasize that subclinical atherosclerosis, as measured by CAC, is prevalent even in rural Indian populations previously presumed to be at lower cardiovascular risk due to traditional lifestyles. These results underscore the need for early cardiovascular risk screening and prevention strategies in rural settings, integrating CAC scoring as a non-invasive tool. This study contributes to global efforts in understanding the ethnic, geographic, and socioeconomic variability in coronary atherosclerosis and reinforces the importance of tailored public health interventions to address the growing cardiovascular burden in India's underserved populations.

## Introduction

Cardiovascular disease (CVD) remains the leading cause of mortality worldwide,^1^^,^^2^ with a significant proportion of individuals with coronary artery disease (CAD) being asymptomatic and unaware of their condition.^3^ In India, the burden of CVD has increased alarmingly over the past decades. As of 2017, CVD accounted for 26.6% of total deaths and 13.6% of total disability-adjusted life years (DALYs), a stark rise from 15.2% to 6.9%, respectively, in 1990.^4^^,^^5^ The India State-Level Disease Burden Study, part of the Global Burden of Disease (GBD) initiative, reported a 2.3-fold increase in the prevalence of ischemic heart disease (IHD) and stroke between 1990 and 2016, with the number of CVD cases nearly doubling from 25.7 million in 1990 to 54.5 million in 2016.^4^ This growing burden is further compounded by high-risk factors such as diabetes and hypertension, with an estimated 72.9 million individuals living with diabetes in 2015 and approximately 207 million people with hypertension as of 2014.^6^ South Asians, particularly those in Indians living in America, are at a higher risk for atherosclerotic cardiovascular disease and IHD-related mortality compared to other ethnic groups.^7^

Coronary artery calcification (CAC), a measure of subclinical atherosclerosis, is an established imaging biomarker and independent predictor of future cardiovascular events. Non-contrast computed tomography (CT) allows non-invasive quantification of CAC, and CAC scoring has been widely utilized in population-based studies to enhance cardiovascular risk prediction beyond traditional risk factors such as hypertension, diabetes, and dyslipidemia.^8^^,^^9^ South Asians over 60 years old have a greater prevalence of coronary artery calcification (CAC) than other racial groups, with its presence being especially pronounced in those with a family history of CAD.^8^^,^^9^ CAC burden varies across racial and ethnic groups, with South Asians living in America exhibiting a higher prevalence and progression of CAC compared to Whites, Blacks, Latinos, and Chinese Americans, as observed in the MESA (Multi-Ethnic Study of Atherosclerosis)^10^^,^^11^ and other cohorts.^12^ However, no studies have assessed CAC burden in Indian adults residing in India to determine its incidence. Moreover, our previous study done in the rural community revealed that nearly one third of the population were at risk of CVDs.^13^

The REACH (Risk Evaluation of Subclinical Coronary Health) Rural India Study is a general population-based prospective study designed to assess the prevalence and burden of CAC among individuals aged 35–65 years in a rural Indian setting. This study incorporates advanced imaging protocols, including CAC scoring, carotid intima-media thickness (cIMT) assessment and ankle-brachial index, alongside comprehensive clinical evaluations. The primary objective of the present study is to determine the prevalence of CAC and its association with cardiometabolic risk factors in a general rural population without previously diagnosed coronary heart disease. To our knowledge, this is the first study from India to establish a cohort aimed at assessing CAC prevalence in the general population.

## Methods

### Study design

**Fig. 1:**
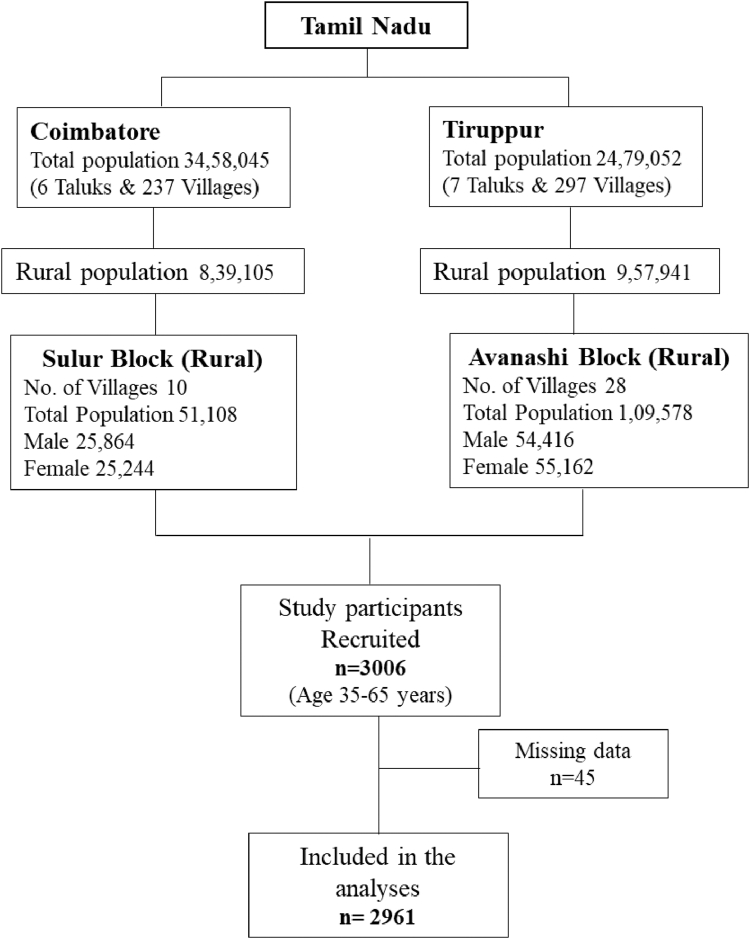
**Flow diagram of the study participants selection methods**.

**Fig. 2:**
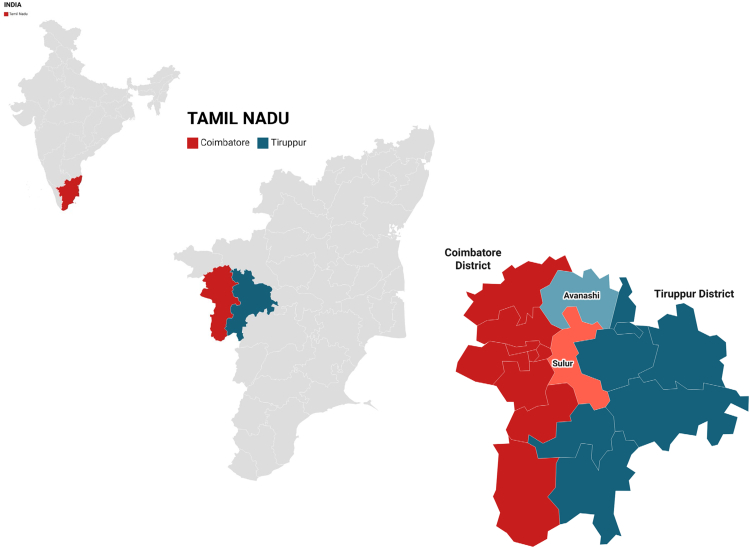
**Map of the sampling areas**.

REACH (Risk Evaluation of Subclinical Coronary Health)-Rural India is a single-center, community-based prospective cohort study conducted in rural areas. From May 2022 to February 2024, men and women aged 35–65 years voluntarily participated from villages surrounding the Coimbatore and Tirupur districts of Tamil Nadu, India (Figs. 1 and 2). We specifically selected rural populations due to the growing burden of noncommunicable diseases in these underserved communities, where subclinical atherosclerotic disease remains poorly characterized. This cohort offers unique insights into coronary artery calcium burden in a population with traditionally lower access to cardiovascular screening and preventive care. This study was conducted as a prospective observational investigation involving a convenience sample of the general rural population. The assessments were chosen for their ability to capture detailed phenotypic characteristics of subclinical disease, while also being feasible for implementation in a large-scale population study.

### Inclusion and exclusion criteria

We applied exclusion criteria similar to those used in MESA.^14^ Participants were excluded if they had a physician-diagnosed myocardial infarction, stroke, transient ischemic attack, heart failure, angina, or were taking statins or antiplatelet drugs. Participants with a history of cardiovascular procedures, such as coronary artery bypass graft surgery, angioplasty, valve replacement, pacemaker or defibrillator implantation, or any heart or arterial surgery, were also excluded. Additionally, individuals with current atrial fibrillation, those undergoing active cancer treatment, and non-native villagers were not included in the study. Twelve-lead electrocardiograms (ECGs) were recorded using a Philips portable ECG device, following the same protocol as the MESA baseline exam. Participants with atrial fibrillation on ECG were excluded from the study.

### Recruitment

We employed a telephone-based recruitment method similar to the MESA^14^ study. A total of 24 villages in and around the Coimbatore and Tirupur districts were selected for participant enrollment (Fig. 1). Before recruitment, the study's purpose, rationale, and design were widely communicated to the target communities. This was achieved through endorsements from community leaders, presentations at community centers, religious and social service organizations, and local awareness campaigns. Additionally, a volunteer team distributed study information sheets door-to-door in households. Initial contact was made by sending informational brochures, inviting recipients to contact the study center by telephone. If we did not receive a response from the villagers, our team visited their residences one or two times to provide reminders in person. Once contact was established and an individual expressed interest in participating, an appointment was scheduled at the designated study center.

Interested individuals contacted the provided phone number, and telephone calls were conducted to identify eligible and willing participants within each household. After a brief screening interview to confirm eligibility and willingness to participate, selected individuals were invited to enroll in the study. Inclusion criteria were verified, and permanent residency was confirmed using government-issued identity cards. Participants were instructed to fast for 12 h before their scheduled study visit. Every Sunday, from May 2022 onward, approximately 30–40 participants were transported to and from Kovai Medical Center and Hospital, Coimbatore from their place of residence.

### Demographic, lifestyle and disease history data from participants

All study visits were conducted by trained bilingual staff, and all consent forms and questionnaires were available in both Tamil and English. We collected comprehensive information on participants, including contact details, demographics, language preferences, tobacco and alcohol use, smoking habits, education, occupation, and lifestyle factors such as physical activity, exercise, dietary preferences (vegetarian or non-vegetarian), junk food consumption (sugary, high salt, processed foods), and sleep duration. Physical activity was assessed based on work-related activities beyond structured exercise. Medical history, healthcare access, family history of cardiovascular diseases (CVDs) and diabetes mellitus (DM), reproductive history (for women), and current prescription and non-prescription medication use, including ayurvedic and herbal supplements, were also documented.

For participants with diabetes or hypertension, disease duration was recorded. Additional information on respiratory issues, chest pain, gestational diabetes, COVID-19 history, COVID vaccination status, and vaccine type were also obtained.

### Anthropometric measurements

Body weight was measured using an electronic weighing scale (SECA 813), while height was recorded with a stadiometer (SECA 208). Waist circumference was measured in centimeters at the end of expiration using a non-stretchable measuring tape, positioned between the costal margins and the iliac crest. Blood pressure was assessed twice, 15 min apart, using an electronic OMRON device (Model HEM-7130, Omron Healthcare, Singapore) on the right arm in a seated position. The average of the two readings was used for analysis. Body mass index (BMI) was calculated using the formula: weight (kg)/height (m^2^).

### Ankle brachial index (ABI)

The Ankle-Brachial Index (ABI) was measured using an OMRON electronic device (Model HEM-8712, Omron Healthcare, Singapore). The participant was positioned supine, and systolic blood pressure readings were taken from the brachial artery, dorsalis pedis artery, and posterior tibial artery on both the left and right sides, following a protocol identical to MESA. ABI was calculated using the formula: right ankle systolic pressure divided by the higher systolic pressure of either arm (left or right). The same formula was applied for the left ankle. The overall ABI was determined as the average of the right and left ABI values.

### Blood collection and assessment of biochemical parameters from blood

Certified phlebotomists collected approximately 10 mL of fasting blood from each participant. Two sets of aliquots were prepared, one for biochemical parameters analysis and the other for storage at −80 °C at the study center. Fasting blood samples were analyzed for various biochemical parameters. Fasting blood glucose was measured using the Hexokinase/GOD-POD/endpoint method. The fasting lipid profile, including total cholesterol, triglycerides, HDL, LDL, and VLDL, was assessed using enzymatic colorimetric tests. Creatinine levels were determined using the Jaffe reaction, while aspartate aminotransferase (AST) and alanine aminotransferase (ALT) were measured using the pyridoxal phosphate-activated liquid reagent test.

All biochemical analyses were conducted using the Roche/Hitachi Cobas 6000 c-501 system (Roche Diagnostics/Hitachi). Estimated glomerular filtration rate (eGFR) was calculated using the 2021 CKD-EPI creatinine equation by the National Kidney Foundation.

### Assessment of carotid intima-media thickness (cIMT)

High-resolution B-mode ultrasonography was performed to measure the intima-media thickness (cIMT) of the right and left internal and common carotid arteries. Carotid ultrasound recordings were conducted at Kovai Medical Center and Hospital using the GE Healthcare Venue 40 system (USA). A trained radiologist identified the carotid bifurcation, differentiated the internal and external carotid arteries, and located the area of maximum wall thickening in the near or far wall, carotid bulb, or internal carotid artery. cIMT was measured at 12 predefined segments (six per side), including one transverse scan of the common carotid artery through the bulb and five longitudinal images from both the right and left carotid arteries. Each image was captured in a specific sequence and recorded for analysis.

### Assessment of CAC

CACS were measured by multi-slice computed tomography (CT) (Siemens Somatom force dual-source CT scanner, Siemens Healthcare, Germany) under fixed 120 kvp and ref mAs of 80 mAs.The scan is prospectively gated with image acquisition during diastole (70% phase). Experienced radiology technicians scanned the heart with z axis coverage from the level of carina to the diaphragm for assessment of coronary calcium deposits. A semi-automated software was used for the calculation of the calcium score (Siemens Syngo Via, Siemens Healthcare, Germany). The calculation was based on the weighted density score given to the highest attenuation value (HU) multiplied by the area of the calcification speck (Agatston score).

Density factor:

• 130–199 HU: 1
• 200–299 HU: 2
• 300–399 HU: 3
• 400+ HU: 4

Based on the density factor, agatston score was calculated. The agatston score ≥1 was identified as having CAC. Further categories were classified based on score as follows:

• 1–10—Minimal Calcification.
• 11–100—Mild calcification,
• 101–400—Moderate calcification,
• >401—Severe calcification.

All the calculations were done by experienced cardiac radiologists. Technicians and radiologists explained the procedure to participants and the importance of breath-hold. ECG electrodes were connected and ensured good connection between electrodes and skin. A stable heart rate was desirable but not mandatory. All the CAC and cIMT images were analyzed independently by two senior radiologists from Kovai Medical Center and Hospital.

### Assessment of covariates

Overweight and obesity were defined as BMI ≥25 kg/m^2^ and ≥30 kg/m^2^,^15^ respectively. Alcohol consumption included daily, weekly, or monthly intake. Smokers encompassed both current and former smokers. Hypertension was defined as a history of drug-induced hypertension or systolic BP ≥ 140 mmHg and/or diastolic BP ≥ 90 mmHg, measured twice 15 min apart in those without a prior diagnosis. Diabetes was classified as a history of diabetes on medication or fasting blood glucose >126 mg/dL. Dyslipidemia was diagnosed by any one of lipid abnormality (total cholesterol >200 mg/dL, triglycerides >200 mg/dL, or LDL cholesterol ≥130 mg/dL, or HDL cholesterol <35 mg/dL in men and <45 mg/dL in females). Elevated creatinine was >1.3 mg/dL in males and >1.1 mg/dL in females. Chronic kidney disease (CKD) was defined as an eGFR <60 mL/min/1.73 m^2^. Peripheral artery disease (PAD) was defined, if ABI average ratio of both right and left ABI ≤0.9. Abnormal cIMT was defined if the right or left cIMT ≥0.1 cm.

### Statistical analysis

Data were initially tabulated using Microsoft Excel and subsequently imported into IBM SPSS Statistics for Windows, Version 29.0 (Armonk, NY) for statistical analysis. Continuous variables were expressed as mean ± standard deviation (SD) or median with interquartile range (IQR), along with 95% confidence intervals (CI) where appropriate. Categorical variables were summarized as frequencies and percentages.

Comparisons between males and females were assessed using independent t test or Mann–Whitney *U* test for continuous variables depending on the distribution, and *χ2* test for categorical variables. Participants were stratified into two groups: those with no coronary artery calcification (No CAC) and those with detectable CAC (CAC ≥1). Socio-demographic characteristics and the prevalence of CAC across metabolic disorders were analyzed using the chi-square test.

For clinical parameters, differences in mean values between the No CAC and CAC groups were evaluated using the independent t-test. Age-stratified comparisons within each sex group were analyzed using one-way analysis of variance (ANOVA) or the Kruskal–Wallis test, based on data distribution. CAC scores typically exhibit a highly right-skewed and zero-inflated distribution, particularly in Asian and South Asian populations,^16^ where a substantial proportion of individuals demonstrate a CAC score of zero despite the presence of cardiometabolic risk factors. This phenomenon, often referred to as the ‘power of zero’ has been consistently reported in large Asian cohorts and reflects delayed calcification, lower plaque calcific density, and potential ethnic differences in atherosclerotic plaque composition compared with Western populations.

To account for this distributional characteristic, CAC values were log-transformed using log (CAC + 1), allowing inclusion of participants with CAC = 0 in parametric analyses while minimizing skewness. In parallel, CAC was categorized into clinically meaningful strata (0, 1–10, 11–100, 101–400, >400) to capture both the presence and severity of coronary calcification. Treating CAC = 0 as a distinct reference category is particularly relevant in Asian populations, where a zero score confers low short-term cardiovascular risk but does not preclude future risk or non-calcified plaque burden. Group-wise comparisons across CAC categories were evaluated within each sex using ANOVA or the Kruskal–Wallis test, as appropriate.

To evaluate the association between metabolic risk factors and the presence of CAC, univariate and multivariate binary logistic regression analyses were conducted. A fully adjusted model included the following covariates: age, sex, BMI, smoking status, hypertension, dyslipidemia, diabetes, overweight, obesity, and non-HDL cholesterol. All regression models were stratified by sex and Age group. Results were reported as odds ratios (ORs) with corresponding 95% confidence intervals (CIs) and p-values.

### Ethics approval

The study was conducted in accordance with the Helsinki Declaration, and the study protocols were approved by the Institutional Ethical Review Board of Kovai Medical Center and Hospital (EC approval no. EC/AP/799/05/2020). Informed consent was obtained from all participants before study procedures commenced.

### Role of the funding source

The funder of the study had no role in the study design, data collection, data analysis, data interpretation, or writing of the report, and had no influence on the decision to submit the paper for publication.

## Results

A total of 3006 participants were recruited for the study from villages in the surrounding districts of Coimbatore and Tirupur, as illustrated in Figs. 1 and 2. Of these, 45 individuals were excluded due to a history of cardiovascular disease (CVD), ongoing cancer treatment, or missing coronary artery calcification (CAC) data. After exclusions, a final sample of 2961 participants was included for further analysis.

### Baseline characteristics and prevalence of coronary artery calcification (CAC)

Table 1 summarizes the baseline characteristics of the 2961 participants (mean age: 49.8 ± 8.3 years), of whom 52.8% were women (n = 1563). The overall prevalence of self-reported diabetes and hypertension was 18.8% and 18.0%, respectively, while 16.3% had a history of COVID-19 positivity. Obesity was present in 12.6% of the population, with a significantly higher prevalence among women than men (16.8% vs. 7.9%; p < 0.001). Abdominal obesity affected 76.0% of participants, and was more prevalent in women (84.6%) compared to men (66.4%; p < 0.001).

**Table 1:** Baseline characteristics of the rural study population stratified by sex.

Parameters	Total	Men (%)	Women (%)	p value
**Sample n (%)**	2961	1398 (47.2)	1563 (52.8)	
**Education**				
Nil	382 (12.9)	84 (6.0)	298 (19.1)	
<5th standard	952 (32.2)	4.3 (28.8)	549 (35.1)	<0.001
<12th standard	128 (43.3)	699 (50.0)	584 (37.4)	
Degree	344 (11.6)	212 (15.2)	132 (8.4)	
**Employment**				
Unemployed	665 (22.5)	41 (2.9)	624 (40.1)	
Farmer	464 (15.7)	223 (16.0)	241 (15.5)	<0.001
Small-scale business	1539 (52)	938 (67.2)	601 (38.6)	
Professional	285 (9.6)	193 (13.8)	92 (5.9)	
Missing	8 (0.3)			
**Food habits**				
Veg	377 (12.7)	162 (11.6)	215 (13.8)	
Non-Veg	2584 (87.3)	1236 (88.4)	1348 (86.2)	0.087
**Vegetables**				
Once a day	1612 (54.4)	799 (57.3)	813 (52.2)	
Twice a day	984 (33.2)	428 (30.7)	556 (35.7)	0.01
Thrice a day	355 (12)	168 (12)	187 (12)	
Missing	10 (0.3)			
**Fruits**				
Daily	651 (22)	266 (19.1)	385 (24.8)	
Weekly	1731 (58.5)	859 (61.8)	872 (56.1)	0.001
Monthly	562 (19)	265 (19.1)	297 (19.1)	
Missing	17 (0.6)			
**Packed food items**				
Daily	196 (6.6)	109 (7.8)	87 (5.6)	
Weekly	688 (23.2)	320 (22.9)	368 (23.5)	
Monthly	1577 (53.3)	733 (52.4)	844 (54)	0.11
Never	500 (16.9)	236 (16.9)	264 (16.9)	
**Meat**				
Daily	28 (0.9)	16 (1.1)	12 (0.8)	
Weekly	1673 (56.5)	818 (58.6)	855 (55)	
Monthly	776 (26.2)	359 (25.7)	417 (26.8)	0.075
Rarely	151 (5.1)	71 (5.1)	80 (5.1)	
Never	323 (10.9)	132 (9.5)	191 (12.3)	
Missing	10 (0.3)			
**Fast food**				
Daily	28 (0.9)	18 (1.3)	10 (0.6)	
Weekly	229 (7.7)	132 (9.5)	97 (6.2)	
Monthly	345 (11.7)	174 (12.5)	171 (11)	0.002
Rarely	905 (30.6)	415 (29.8)	490 (31.5)	
Never	1442 (48.7)	655 (47)	787 (50.6)	
Missing	12 (0.4)			
**Soft drinks**				
Yes	1033 (34.9)	515 (36.8)	518 (33.1)	
No	1928 (65.1)	883 (63.2)	1045 (66.9)	0.03
**Physical activity**				
Vigorous	353 (11.9)	207 (14.8)	146 (9.3)	
Moderate	1598 (54)	764 (54.6)	834 (53.4)	<0.001
Less	1010 (34.1)	427 (30.5)	583 (37.3)	
**Exercise**				
0–0.5 h	2190 (74)	997 (71.3)	1193 (76.3)	
0.6–1.0 h	506 (17.1)	276 (19.7)	230 (14.7)	0.001
1.1–3.0 h	149 (5.0)	77 (5.5)	772 (4.6)	
>3.0 h	116 (3.9)	48 (3.4)	68 (4.4)	
**Alcohol**				
Daily	52 (1.8)	52 (3.7)	–	
Weekly	269 (9.1)	269 (19.2)	–	
Monthly	138 (4.7)	138 (9.9)	–	
Rarely	181 (6.1)	181 (12.9)	–	
Never	2321 (78.4)	758 (54.2)	–	
**Smoking**				
Current	287 (9.7)	287 (20.6)	–	
Ex-smoker	164 (5.5)	164 (11.8)	–	
Non-smoker	2502 (84.5)	939 (67.6)	–	
**Sleep duration**				
10–12 h	143 (4.8)	72 (5.2)	71 (4.5)	
7–9 h	1767 (59.7)	857 (61.3)	910 (58.2)	
4–6 h	906 (30.6)	414 (29.6)	492 (31.5)	0.055
<4.0 h	145 (4.9)	55 (3.9)	90 (5.8)	
**Known T2DM**				
Yes	557 (18.8)	309 (22.1)	248 (15.9)	<0.001
No	2404 (81.2)	1089 (77.9)	1315 (84.1)	
**Known HTN**				
Yes	534 (18)	272 (19.5)	262 (916.8)	0.06
No	2427 (82)	1126 (80.5)	13.1 (83.2)	
**Family history of CAD**				0.950
Yes	277 (9.4)	130 (9.4)	147 (9.5)	
No	2657 (89.7)	1253 (90.6)	1404 (90.5)	
Not known	27 (0.9)			
**History of Covid**				
Yes	482 (16.3)	265 (19)	217 (13.9)	<0.001
No	2479 (83.7)	1133 (81)	1346 (86.1)	
**Overweight**	1369 (46.2)	610 (43.6)	759 (48.6)	<0.001
**Obesity**	373 (12.6)	110 (7.9)	263 (16.8)	<0.001
**Abdominal obesity**	2251 (76)	928 (66.4)	1323 (84.6)	<0.001
**Hypertension**	1145 (38.7)	582 (41.6)	563 (36)	0.001
**Diabetes**	736 (24.9)	404 (28.9)	332 (21.2)	<0.001
**Dyslipidemia**	2456 (82.9)	1173 (83.9)	1283 (82.1)	0.103
**Non-HDL**	1920 (64.8)	919 (65.7)	1001 (64)	0.178
**High LDL**	1392 (47)	655 (46.9)	737 (47.2)	0.45
**Abnormal cIMT**	150 (5.1)	103 (7.4)	47 (3)	<0.001
**PAD**				
**Moderate**	828 (28)	236 (16.9)	592 (37.9)	<0.001
**Severe**	196 (6.6)	34 (2.4)	162 (10.4)	
**CAC category**				
**Any CAC**	757 (25.6)	468 (33.5)	282 (18.5)	<0.001
**CAC score 0**	2204 (74.4)	930 (66.5)	1274 (81.5)	
**CAC score 1**–**10**	208 (7)	122 (8.7)	86 (5.5)	
**CAC score 11**–**100**	339 (11.4)	212 (15.2)	127 (8.1)	
**CAC score 101**–**400**	154 (5.2)	93 (6.7)	61 (3.9)	
**CAC score >400**	56 (1.9)	41 (2.9)	15 (1)	<0.001
**Multiple risk factor**				
**0**	558 (18.8)	209 (14.9)	349 (22.3)	
**1**	1276 (43.1)	610 (43.6)	666 (42.6)	
**2**	856 (28.9)	442 (31.6)	414 (26.5)	
**3**	271 (9.2)	137 (9.8)	134 (8.6)	<0.001

This table presents the sociodemographic, lifestyle, dietary, and clinical characteristics of the study participants (N = 2961), stratified by sex. Categorical variables are reported as counts and percentages. Statistical comparisons between men and women were performed using the chi-square test for categorical variables. Multiple risk factors-participants were categorized by number of risk factors (0, 1, 2, ≥3 risk factors) (diabetes, hypertension and non-HDL). A p-value <0.05 was considered statistically significant.

The prevalence of diagnosed hypertension was 38.7%, with a higher proportion observed in men than women (41.6% vs. 36.0%; p = 0.001). Similarly, diabetes was significantly more common among men (28.9%) than women (21.2%; p < 0.001). Conversely, dyslipidemia (82.9%), elevated non-HDL cholesterol (64.8%), and high LDL cholesterol (47.0%) showed no significant sex differences (p > 0.05). Markers of subclinical atherosclerosis showed sex-specific differences. Abnormal carotid intima–media thickness (cIMT) was observed in 5.1% of the total population, significantly more in men than women (7.4% vs. 3.0%; p < 0.001). Peripheral arterial disease (PAD) was notably more prevalent among women, with moderate PAD seen in 37.9% and severe PAD in 10.4%, compared to 16.9% and 2.4% in men, respectively (p < 0.001).

Coronary artery calcium (CAC) was detectable (CAC score ≥1) in 25.6% of participants, with a higher prevalence in men than women (33.5% vs. 18.5%; p < 0.001). Most participants (74.4%) had no detectable CAC. The distribution of CAC severity was as follows: mild CAC (1–10 AU) in 7.0%, moderate (11–100 AU) in 11.4%, advanced (101–400 AU) in 5.2%, and severe (>400 AU) in 1.9%. The sex-specific breakdown of CAC scores was:

Men: 1–10 AU (8.7%), 11–100 AU (15.2%), 101–400 AU (6.7%), >400 AU (2.9%).

Women: 1–10 AU (5.5%), 11–100 AU (8.1%), 101–400 AU (3.9%), >400 AU (1.0%).

Risk factor clustering analysis revealed that 18.8% of participants had no conventional cardiometabolic risk factors, 43.1% had one, 28.9% had two, and 9.2% had three or more. The presence of multiple risk factors (≥2) was significantly more common among men (p < 0.001).

Table 2 presents detailed sex-wise comparisons of clinical and biochemical characteristics. Men were slightly older than women (mean age: 50.09 ± 7.89 vs. 48.54 ± 7.86 years; p < 0.001). Significant sex-based differences were observed across multiple clinical parameters including body mass index (BMI), waist circumference (WC), systolic and diastolic blood pressure, fasting blood glucose, serum creatinine, total cholesterol, triglycerides, HDL cholesterol, AST, ALT, serum uric acid, ankle–brachial index (ABI), CAC scores, and cIMT (all p < 0.001). In age-adjusted analyses, HDL cholesterol levels were not significantly different between sexes (see Supplementary Table S1). Among participants with no coronary calcium, the median age was 47 years. Women had a higher proportion of CAC = 0 (81.6%) compared to men. Sex-stratified analysis of CAC categories revealed that in men, there were no significant differences in total cholesterol, triglycerides, HDL cholesterol, uric acid, or ABI ratio across CAC severity groups. Among women, no significant differences were observed across CAC categories for BMI, serum creatinine, AST, ALT, or ABI ratio (Supplementary Table S2).

**Table 2:** Clinical characteristics of study population by sex.

Parameters	Total (n = 2961)	Men (n = 1398)	Women (n = 1563)	p value
Age	49.27 (7.919) [48.99–49.56]	50.09 (7.89) [49.67–50.5]	48.54 (7.86) [48.15–48.93]	<0.001
BMI	26.2 (6.1) [26.0–26.44]	25.41 (4.1) [25.19–25.63]	26.94 (7.43) [26.94–26.58]	<0.001
WC	93.08 (11.01) [92.68–93.48]	94.88 (10.61) [94.32–95.44]	91.49 (11.12) [91.49–90.93]	<0.001
SBP	126.85 (18.78) [126.17–127.53]	128.369 (17.790) [127.43–129.30]	125.50 (19.54) [125.50–124.53]	<0.001
DBP	78.82 (12.3) [78.38–79.27]	80.14 (12.11) [79.50–80.77]	77.65 (12.41) [77.65–77.04]	<0.001
FBG	111.08 (44.81) [111.07–109.46]	114.289 (47.130) [111.81–116.75]	108.21 (42.45) [108.21–106.10]	<0.001
Creatinine	0.73 (0.26) [0.72–0.74]	0.84 (0.27) [0.83–0.85]	0.63 (0.21) [0.63–0.62]	<0.001
TC	186.14 (38.34) [184.75–187.52]	184.28 (37.435) [182.3–186.25]	187.80 (39.24) [187.80–185.85]	0.013
TG	137.75 (104.69) [133.98–141.53]	151.49 (105) [145.98–157.00]	125.46 (102.91) [125.46–120.36]	<0.001
L-HDL	40.68 (9.1) [40.35–41.01]	37.93 (8.66) [37.47–38.38]	43.14 (8.86) [43.14–42.70]	<0.001
H-LDL	129.33 (34.69) [128.0–130.58]	128.60 (34.81) [126.77–130.42]	129.98 (34.58) [129.98–128.26]	0.28
AST	21.08 (10.34) [20.71–21.45]	22.80 (12.62) [22.14–23.46]	19.54 (7.45) [19.54–19.17]	<0.001
ALT	19.93 (13.3) [19.45–20.41]	23.33 (15.51) [22.51–24.14]	16.89 (10.03) [16.39–17.39]	<0.001
Uric acid	5.16 (10.5) [4.79–5.54]	5.41 (1.40) [5.34–5.49]	4.43 (1.23) [4.37–4.49]	<0.001
ABI ratio	1.0 (0.07) [1.00–1.01]	1.03 (0.05) [1.02–1.03]	0.99 (0.07) [0.99–0.98]	<0.001
CAC	32.81 (163.51) [26.91–38.70]	47.64 (203.81) [36.94–58.33]	19.54 (114.63) [19.54–13.86]	<0.001
cIMT (cm) right	0.06 (0.03) [0.05–0.06]	0.06 (0.02) [0.060–0.062]	0.06 (0.03) [0.06–0.06]	<0.001
cIMT (cm) left	0.06 (0.02) [0.061–0.063]	0.064 (0.02) [0.063–0.066]	0.06 (0.03) [0.06–0.06]	<0.001

This table presents the baseline characteristics of the study participants (N = 2961), stratified by sex. Continuous variables are reported as mean (SD) or Median (IQR) with 95% confidence intervals in parentheses. Categorical variables are reported as counts and percentages. Statistical comparisons between men and women were performed using the independent t-test for continuous variables and the chi-square test for categorical variables. Systolic blood pressure (SBP), diastolic blood pressure (DBP), fasting blood glucose (FBG), total cholesterol (TC), triglycerides (TG), low-density lipoprotein cholesterol (LDL-C), high-density lipoprotein cholesterol (HDL-C), aspartate aminotransferase (AST), alanine aminotransferase (ALT), serum uric acid, serum creatinine, waist circumference (WC), body mass index (BMI), and ankle-brachial index (ABI). Multiple risk factors-participants were categorized by number of risk factors (0, 1, 2, ≥3 risk factors) (diabetes, hypertension and non-HDL). A p-value <0.05 was considered statistically significant.

**Fig. 3:**
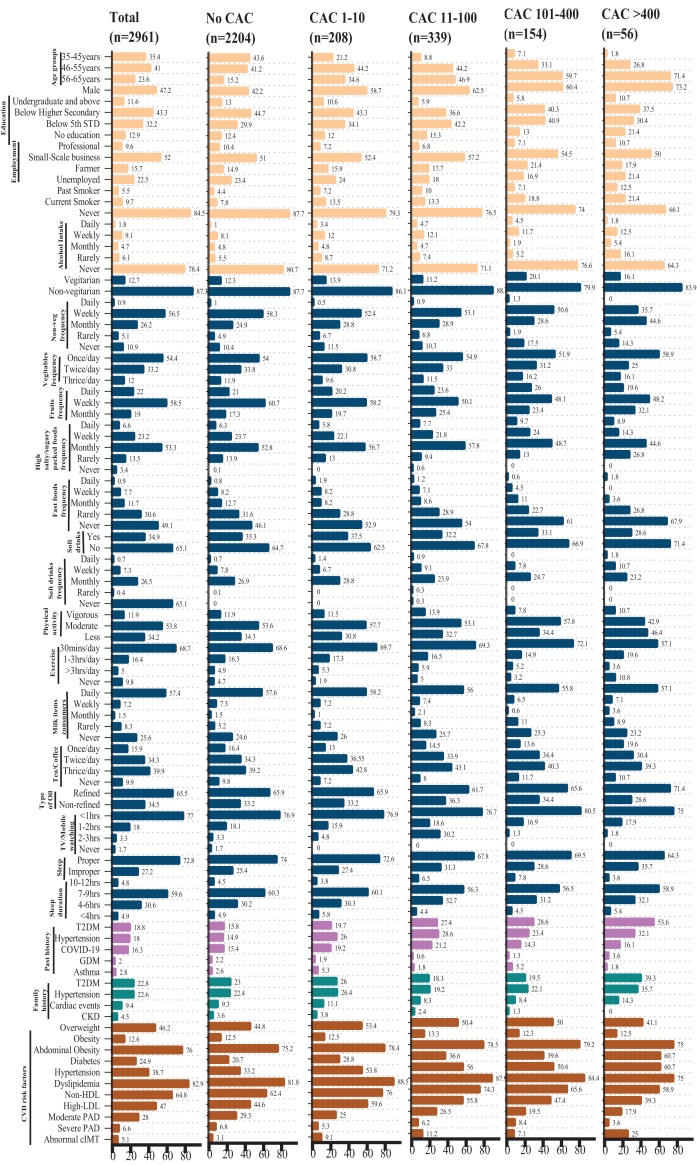
**Participants demography, lifestyle, medical history and metabolic diseases by CAC categories**.

Participants characterized by different CAC categories are shown in Fig. 3. The largest proportion of participants (41.0%) belonged to the 46–55 years age group, followed by 35.4% in the 35–45 years group and 23.6% in the 56–65 years group (p < 0.001) (Fig. 3).

Group 1 (No CAC): The prevalence of hypertension (33.2%), diabetes (20.7%), and elevated cIMT (3.1%) was relatively lower than in other CAC groups.

Group 2 (CAC 1–10): Participants with minimal CAC accounted for about 7% of the cohort.

The proportion of men (58.7%) was higher in this group. The prevalence of hypertension nearly doubled (53.8%), while diabetes increased to 28.8% compared with the No-CAC group.

Group 3 (CAC 11–100): In the moderate CAC range, participants demonstrated a clear clustering of metabolic abnormalities, with men comprising 62.5% of this group.

Group 4 (CAC 101–400): Participants with moderately high CAC scores were predominantly men (60.4%).

Group 5 (CAC >400): This group represents individuals with severe CAC and advanced metabolic dysregulation. They constituted approximately 2% of the cohort, with an overwhelming men predominance (73.2%). This subgroup exhibited the highest prevalence of hypertension (60.7%), diabetes (60.7%), and elevated cIMT (25%). Current smoking was reported by 21.4% (Fig. 3).

Among men, median CAC scores (50th percentile) were zero in the 35–45 and 46–55 age groups but increased to 11.55 AU in those aged 56–65 years. The 75th and 90th percentiles showed a marked rise with age, reaching 85.97 AU and 337.4 AU respectively in the oldest group (Table 3). In women, CAC scores remained zero at the 50th percentile across all age groups (Table 3). However, higher percentiles began to show detectable CAC in older age groups, with the 75th percentile reaching 36.15 AU and the 90th percentile 165.38 AU in the 56–65 age group (Table 3).

**Table 3:** Coronary artery calcium score percentiles for men and women within age strata.

Age group	Men			Women		
	35–45 (n = 432)	46–55 (n = 592)	56–65 (n = 374)	35–45 (n = 615)	46–55 (n = 623)	56–65 (n = 325)
CAC percentiles (AU)						
25th	0	0	0	0	0	0
50th	0	0	11.55	0	0	0
75th	0	5.77	85.97	0	0	36.15
90th	3.5	74.36	337.4	0	24.8	165.38

AU, Agatston units.

### Prevalence of CAC and other metabolic risk factors

**Fig. 4:**
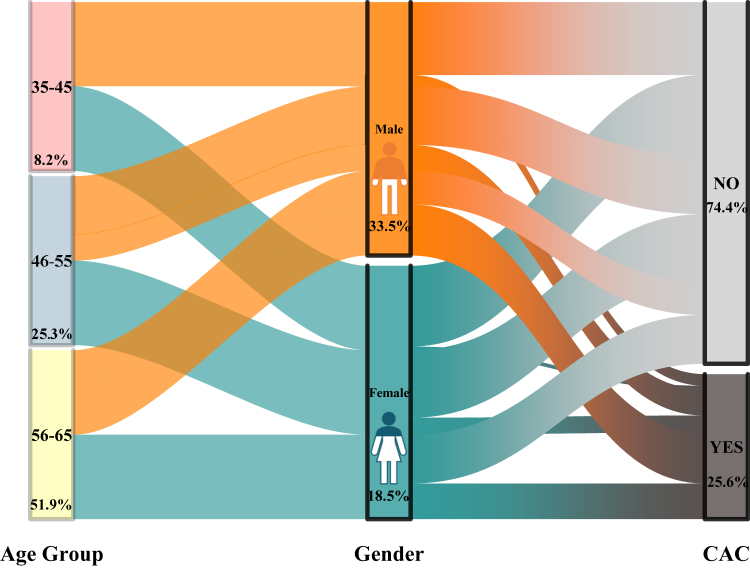
Prevalence of coronary artery calcification stratified by age group and gender.

**Fig. 5:**
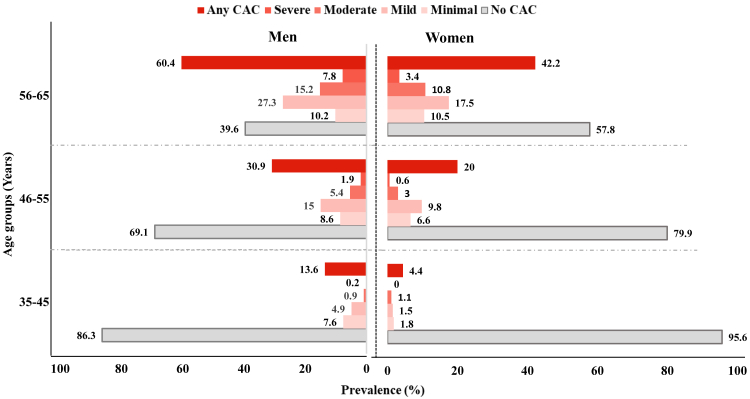
**Age and sex specific prevalence of CAC. No calcification—0, Minimal 1–10, Mild 11–100, Moderate 101–400, Severe >400**.

The overall prevalence of coronary artery calcification (CAC) in the study population was 25.6% (Fig. 4). A significant sex-based disparity was observed, with men exhibiting a higher prevalence of CAC than women (33.5% vs. 18.5%). Age-stratified analysis revealed that the burden of CAC increased with age, with the highest prevalence observed among participants aged 56–65 years (51.9%), followed by those aged 46–55 years (25.3%), and 35–45 years (8.2%) (Fig. 4). Across all age categories, the prevalence of CAC categories was consistently higher in men (Fig. 5). Importantly, no severe CAC (>400) was observed among women in the 35–45year age group. Among men, the prevalence of any CAC (≥1) was 13.6%, 30.9%, and 60.4% in the age groups of 35–45, 46–55, and 56–65 years, respectively; whereas among women, it was 4.4%, 20.0%, and 42.2% in the corresponding age groups (Fig. 5).

Table 4 presents the distribution of key metabolic risk factors among participants stratified by CAC status. The overall prevalence of abdominal obesity was high (76%) and showed no significant difference between groups with and without CAC (p = 0.12). However, overweight (50.4%) and obesity (12.8%) were more frequently observed among participants with any CAC (p = 0.013). Hypertension and diabetes were notably more prevalent in the CAC group compared to those without CAC, with 54.6% and 33.1% affected respectively (both p <0.001). Although the prevalence of elevated creatinine (0.7%) and reduced eGFR (<60 mL/min/1.73 m²) (0.6%) was relatively low, the difference in eGFR reached statistical significance (p = 0.036). Dyslipidemia was highly prevalent in the cohort (82.9%) and significantly more common in the any CAC group (p < 0.001). Similarly, abnormal carotid intima-media thickness (cIMT) was observed more frequently in those with CAC (p < 0.001). While severe peripheral artery disease (PAD) was seen in 6.7% of participants and more commonly among those without CAC. Moderate PAD affected 28% of the population and did not differ significantly between the CAC groups (p = 0.11).

**Table 4:** Prevalence of metabolic risk factors among participants stratified by Coronary Artery Calcification (CAC).

Parameters	Total (n = 2961)	No CAC (n = 2204)	Any CAC (n = 757)	p value
Abdominal obesity	2251 (76)	1658 (75.2)	593 (78.3)	0.12
Overweight	1369 (46.2)	987 (44.7)	382 (50.4)	0.013
Obesity	373 (12.6)	276 (12.5)	97 (12.8)	
Hypertension	1145 (38.8)	731 (33.1)	414 (54.6)	<0.001
Diabetes	737 (24.9)	457 (20.7)	280 (36.9)	<0.001
Elevated creatinine	21 (0.7)	12 (0.5)	9 (1.1)	0.079
eGFR of <60 mL/min/1.73 m^2^	19 (0.6)	10 (0.4)	9 (1.1)	0.036
Dyslipidaemia	2456 (82.9)	1802 (81.7)	654 (86.3)	<0.001
Severe PAD	196 (6.6)	149 (6.7)	47 (6.2)	
Moderate PAD	828 (28)	646 (29.3)	182 (24.0)	0.11
Abnormal cIMT	150 (5.1)	68 (3.0)	82 (10.8)	<0.001

Values are n (%). No CAC (score = 0) and Any CAC (score ≥1).

### Association between metabolic risk factors and presence of Coronary Artery Calcification (CAC)

**Fig. 6:**
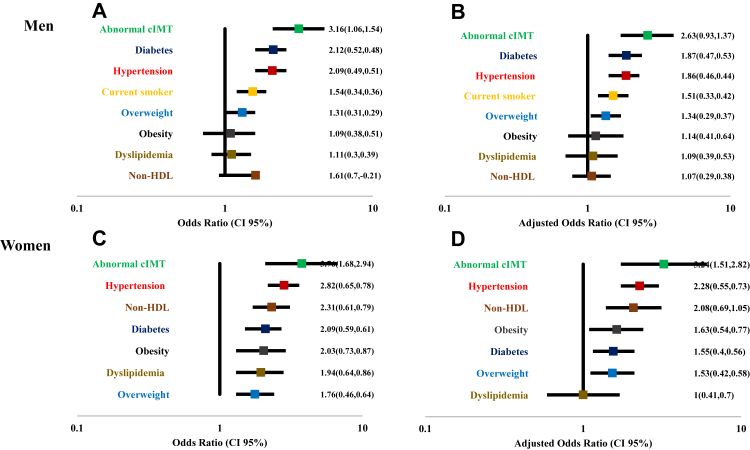
**Association between metabolic risk factors and presence of CAC. (Adjusted with diabetes, dyslipidemia, overweight, obesity, cIMT, hypertension, current smoker, non-HDL)**. A, Men (unadjusted model); B, Men (adjusted model); C, Women (unadjusted model); D, Women (adjusted model). Adjusted models include relevant covariates.

The multivariate regression analysis revealed several sex-specific associations between metabolic risk factors and the presence of CAC (Fig. 6). In men, diabetes (OR: 1.87; 95% CI: 1.4–2.4; p < 0.001), overweight (OR: 1.34; 95% CI: 1.05–1.71; p = 0.016), abnormal carotid intima-media thickness (cIMT) (OR: 2.63; 95% CI: 1.7–4.0; p < 0.001), hypertension (OR: 1.86; 95% CI: 1.4–2.3; p < 0.001), and current smoking (OR: 1.51; 95% CI: 1.18–1.93; p = 0.001) remained significantly associated with CAC after full adjustment Obesity, dyslipidemia, and non-HDL cholesterol were not significantly associated with CAC in men.

Among women, fully adjusted models showed that diabetes (OR: 1.55; 95% CI: 1.15–2.11; p = 0.004), overweight (OR: 1.53; 95% CI: 1.11–2.11; p = 0.008), obesity (OR: 1.63; 95% CI: 1.09–2.4; p = 0.017), abnormal cIMT (OR: 3.24; 95% CI: 1.73–6.06; p < 0.001), hypertension (OR: 2.28; 95% CI: 1.73–3.01; p < 0.001), and elevated non-HDL cholesterol (OR: 2.08; 95% CI: 1.39–3.13; p < 0.001) were all significantly associated with the presence of CAC. Notably, dyslipidemia lost its significance after adjustment in women (Fig. 6).

Age-stratified multinomial logistic regression revealed marked heterogeneity in metabolic determinants of CAC across age groups (see Supplementary Table S3). In participants aged 35–45 years, hypertension, overweight/obesity, and current smoking were significantly associated with CAC in unadjusted analyses; however, none retained independent significance after adjustment, with only borderline associations observed for hypertension and smoking. The 46–55-year age group demonstrated the strongest and most consistent independent associations. After multivariable adjustment, diabetes (OR 1.72; 95% CI: 1.27–2.32), hypertension (OR 1.65; 95% CI: 1.25–2.18), overweight (OR 1.56; 95% CI: 1.09–2.24), abnormal IMT (OR 1.92; 95% CI: 1.05–3.52), current smoking (OR 1.55; 95% CI: 1.06–2.27), and elevated non-HDL cholesterol (OR 1.53; 95% CI: 1.06–2.27) were independently associated with CAC. Among individuals aged 56–65 years, diabetes was the only risk factor independently associated with CAC (OR 1.42; 95% CI: 1.02–1.91), while associations with other metabolic factors were attenuated after adjustment.

## Discussion

In this general population-based study of 2961 adults from rural South India, we observed a 25.6% overall prevalence of coronary artery calcification (CAC) among asymptomatic adults aged 35–65 years. The burden was significantly higher in men (33.5%) than in women (18.5%), and increased markedly with increasing age. These findings contribute valuable data to the global understanding of subclinical atherosclerosis, particularly from underrepresented low-resource rural populations in South Asia. Our study is unique in its focus on lower-middle-income rural communities with limited access to cardiovascular screening, where CAC imaging is rarely implemented.

A consistent and graded increase in cardiometabolic risk factors was observed across CAC categories. Advancing CAC scores were associated with older age, a higher proportion of men, and a progressive rise in the prevalence of hypertension, diabetes, and obesity. Likewise, current smoking, alcohol consumption, elevated LDL-cholesterol, and abnormal carotid intima-media thickness (cIMT) increased steadily with CAC severity. Participants with higher CAC categories also showed a higher frequency of elevated cIMT, reflecting systemic atherosclerotic involvement. In contrast, individuals with no detectable CAC exhibited the lowest burden of metabolic abnormalities and healthier lifestyle profiles. These findings indicate a clear dose–response relationship between cumulative cardiometabolic risk and subclinical coronary calcification, highlighting CAC as a robust marker of vascular aging and cardiometabolic disease progression in this population.

Our percentile data provide new insights into the burden of CAC in a rural Indian cohort. The 75th percentile of CAC was zero in women up to age 55, and in men, it remained zero up to age 45, but increased to 5.77 AU in the 46–55 age group and 85.97 AU in those aged 56–65 years. Similarly, the 90th percentile CAC value in men aged 56–65 years was 337.4 AU, while in women of the same age range, it was 165.38 AU. In comparison, the MESA^10^ study found the 75th percentile at age 45 to be CAC = 0 for all racial groups. The 90th percentile at age 45 in MESA was 16 AU for Black males and 36 AU for White males,^10^ whereas in our study, the 90th percentile CAC in men at age 45 was 3.5 AU. The higher burden of CAC in men compared to women aligns with observations from large multi-ethnic studies such as MESA^10^ and MASALA.^9^ Notably, severe CAC (Agatston score >400) was nearly threefold more prevalent in older men (56–65 years), suggesting a cumulative exposure to atherogenic risk factors.

The CAC prevalence (25.6%) among asymptomatic rural Indian adults aligns remarkably well with global population-based findings from high-income and urban settings. Notably, the MASALA study demonstrated that South Asian men living in US exhibited CAC prevalence and progression rates comparable to White American men and significantly higher than those observed in Black, Latino, and Chinese American men, even after adjusting for traditional cardiovascular risk factors.^11^ South Asian men living in US demonstrate a higher prevalence of CAC as compared to South Asian women living in US (55.9% vs. 22.7%).^11^ Our rural cohort showed the prevalence of CAC in men higher than women (33.5% vs. 18.5%). These findings, along with our results, provide converging evidence that South Asians, particularly men, may exhibit a comparable or higher susceptibility to subclinical atherosclerosis despite having differing traditional risk profiles. The MASALA and MESA studies also caution that differences in CAC assessment methods and population characteristics can make direct comparisons challenging.^9^^,^^10^ Nonetheless, the consistent detection of CAC across these cohorts emphasizes the biological relevance and cross-ethnic applicability of CAC as a marker of coronary atherosclerosis. It is challenging to compare changes in CAC across studies because different methods of study design have been used (see Supplementary Table S4).

Our findings reaffirm that metabolic risk factors, particularly diabetes, hypertension, dyslipidemia, overweight, and obesity are significantly associated with CAC development. These associations persisted after adjustment for age, sex, and BMI, aligning with observations from the Heinz Nixdorf Recall,^17^ DANCAVAS,^18^ and MASALA^9^ cohorts. The high burden of noncommunicable diseases (NCDs) in rural farming communities further underscores the need for early cardiovascular risk screening to prevent undiagnosed subclinical atherosclerosis from progressing into overt cardiovascular events.^13^^,^^19^

Interestingly, smoking in men demonstrated a strong association with CAC, while alcohol consumption did not. This may be attributable to low overall alcohol intake and potential underreporting due to cultural norms. These findings are consistent with prior evidence showing stronger and more consistent links between smoking and vascular calcification than alcohol, particularly in male-dominated settings.^20^ Because women in this cohort largely abstain from alcohol and smoking, these variables were assessed only in men.

This study demonstrates that the association between metabolic risk factors and coronary artery calcification (CAC) is both age-dependent and sex-specific, with the strongest and most consistent effects observed during midlife and differing patterns between men and women. Age-stratified analyses identified 46–55 years as a critical transition period in which multiple metabolic and vascular risk factors independently contributed to CAC. Diabetes and hypertension emerged as the most robust predictors, accompanied by overweight, smoking, elevated non-HDL cholesterol, and abnormal carotid IMT. In contrast, among younger adults (35–45 years), several risk factors showed associations only in unadjusted models, suggesting early clustering of cardiometabolic risk without established coronary calcification,^21^ while in older adults (56–65 years), diabetes remained the sole independent determinant, likely reflecting cumulative exposure and survivor or statin therapy effects.^22^

Sex-stratified analyses further revealed distinct biological and risk profiles. In men, CAC was independently associated with diabetes, hypertension, overweight, smoking, and abnormal cIMT, indicating a dominant role of traditional cardiometabolic risk factors and lifestyle exposures. In women, however, CAC showed strong independent associations with diabetes, hypertension, overweight, obesity, non-HDL cholesterol, and abnormal cIMT, with effect sizes for hypertension and cIMT exceeding those observed in men. However, obesity and atherogenic dyslipidaemia showed stronger associations with coronary calcification in women in our cohort, indicating that women with metabolic dysfunction (e.g., diabetes) often have a greater relative cardiovascular risk compared to men,^23^ potentially reflecting sex-specific metabolic and hormonal influences on atherosclerotic pathways.^24^ Women's cardiovascular risk accelerates around the menopausal transition, accompanied by adverse lipid changes and loss of estrogen-related vascular protection, which may amplify the impact of obesity and dyslipidaemia on subclinical atherosclerosis.^24^ Conversely, smoking remains a potent risk factor across sexes, and some large cohort studies have reported particularly elevated cardiovascular risk associated with smoking in women,^25^ consistent with differential behavioral exposure patterns and susceptibility.

A significant and independent association between abnormal carotid intima-media thickness (cIMT) and CAC was observed in both men and women. This reinforces the shared pathophysiological basis of carotid and coronary atherosclerosis, corroborating findings from MESA,^26^ DANCAVAS,^18^ and BioImage^27^ studies. In contrast, ankle-brachial index (ABI) as a marker of peripheral artery disease (PAD) did not demonstrate a significant association with CAC in our population. This diverges from studies such as MESA,^28^ where PAD and CAC often coexisted, and may reflect differences in disease patterns, vascular remodeling, or measurement sensitivity in rural Indian populations. While reduced estimated glomerular filtration rate (eGFR) showed an association with CAC in unadjusted models, this link was attenuated after adjusting for age, sex, and BMI. This is consistent with findings from asymptomatic Brazilian cohorts, where eGFR was not independently associated with CAC.^29^

Emerging evidence underscores that individuals with subclinical atherosclerosis especially those with low or zero CAC scores may still be at risk for disease progression. A LASSO-based predictive nomogram by Liang et al. (2025), identified key predictors, including hypertension, HbA1c, and body fat percentage, even among double-zero CAC patients. These findings reinforce the importance of early, individualized intervention. In our study, these same risk factors hypertension, diabetes, obesity were strongly associated with increasing CAC burden. Preventing CAC progression in such populations should prioritize blood pressure and glucose control, weight management, and lifestyle modification (diet, physical activity, smoking cessation). Combining risk prediction tools with tailored cardiovascular education may enhance preventive strategies before clinical events manifest.^30^

This study provides a detailed CAC-based vascular risk profiling in a rural Indian population, integrating metabolic, vascular, and biochemical assessments. Strengths include a large, community-based cohort, standardized CT-based CAC scoring, and comprehensive risk factor profiling. However, several limitations must be acknowledged. First, the study employed convenience sampling rather than random selection due to logistical constraints, which may introduce selection bias and potentially overestimate CAC prevalence. Second, fasting blood glucose was used for diabetes diagnosis instead of HbA1c, which might underestimate chronic glycemic burden. The cross-sectional design limits the ability to assess CAC progression or future cardiovascular events. In addition, dietary patterns and physical activity were not comprehensively assessed in the present study and may act as residual confounders in the observed associations between metabolic risk factors and coronary artery calcification. Although the study included a large, community-based sample, participants were recruited from selected rural regions, which may limit the generalizability of the findings to the broader Indian population, especially urban and high-income groups.

This study comprehensively characterized CAC prevalence and its association with metabolic and vascular risk factors in a rural Indian population. Our results highlight a significant burden of subclinical atherosclerosis, particularly in middle-aged men, and underscore the importance of early detection and prevention strategies for cardiovascular disease in underserved populations. Further longitudinal studies are warranted to assess CAC progression and validate its predictive value for clinical cardiovascular events in rural India. These results could help reform rural Indian health policies for screening of sub-clinical atherosclerosis in these undeserved groups and enhance the prediction and prevention of CVDs in these groups.

## Contributors

**Conceptualisation:** Thomas Alexander, Mohanraj Sundaresan, Krishnan Swaminathan, Arulraj Ramakrishnan.

**Data curation:** Thomas Alexander, Mohanraj Sundaresan, Avinash Kumar Ragupathy.

**Formal analysis:** Thomas Alexander, Mohanraj Sundaresan, Avinash Kumar Ragupathy, Pudhiavan A, Manoj Kumar B, Dharam J. Kumbhani.

**Funding acquisition:** Thomas Alexander.

**Investigation:** Thomas Alexander, Mohanraj Sundaresan, Avinash Kumar Ragupathy, Arulraj Ramakrishnan, Jeevithan, Velmurugan, Mathew Cherian, Pudhiavan.

**Methodology:** Thomas Alexander, Mohanraj Sundaresan, Avinash Kumar Ragupathy, Velmurugan, Dharam J. Kumbhani, Haritha Thambi BS, Parthasarathy A, Karthika D, Samrat Ashok V, Srinidhi Narayani S, Buvaneswari G, Gowtham M, Madhavi S, Gowri S, Balakumaran.

**Project administration:** Thomas Alexander, Mohanraj Sundaresan.

**Resources:** Mohanraj Sundaresan, Avinash Kumar Ragupathy, Velmurugan, Haritha Thambi BS, Parthasarathy A, Karthika D, Samrat Ashok V, Srinidhi Narayani S, Buvaneswari G, Gowtham M, Madhavi S, Gowri S.

**Supervision:** Thomas Alexander, Mohanraj Sundaresan, Arulraj Ramakrishnan.

**Validation:** Thomas Alexander, Mohanraj Sundaresan, Arulraj Ramakrishnan, Mathew Cherian.

**Writing:** Thomas Alexander, Mohanraj Sundaresan, Avinash Kumar Ragupathy.

Authors have directly accessed and verified data reported in the manuscript: Thomas Alexander, Mohanraj Sundaresan, Avinash Kumar Ragupathy, Krishnan Swaminathan, Puthiavan, Mathew Cherian, Arulraj Ramakrishnan, Dharam J. Kumbhani.

**Thomas Alexander** and **Mohanraj Sundaresan** had full access to all the data in the study and take responsibility for the integrity of the data and the accuracy of the data analysis.

All authors contributed substantially to the conception and design of the study, data analysis, and interpretation. All authors reviewed the manuscript critically for important intellectual content, contributed to revisions, and approved the final version for submission.

## Data sharing statement

Deidentified individual participant data and the data that underlie the results reported in this article will be made available upon reasonable request to the corresponding author, following institutional and ethical approval, for research purposes that are in line with the study objectives.

## Editor note

The Lancet Group takes a neutral position with respect to territorial claims in published maps and institutional affiliations.

## Declaration of interests

The authors declare that they have no conflict of interests.
