# Supplementary material for: Burden of subclinical coronary atherosclerosis among asymptomatic adults: the REACH–Rural India Study
Source: Lancet Reg Health Southeast Asia. 2026 Feb 11;45:100726. doi: 10.1016/j.lansea.2026.100726 (PMC12915248; doi:10.1016/j.lansea.2026.100726)
Supplement: Supplementary Tables [file mmc1.doc]

**Supplementary Table 1:** Clinical and biochemical parameters stratified by age group and sex

| **Parameters** | **Men** | | | | | | | **Women** | | | | | |  |
| --- | --- | --- | --- | --- | --- | --- | --- | --- | --- | --- | --- | --- | --- | --- |
| **Age group** | **35-40** | **41-45** | **46-50** | **51-55** | **56-60** | **61-65** | **p Value** | **35-40** | **41-45** | **46-50** | **51-55** | **56-60** | **61-65** | **p Value** |
| **Sample, n (%)** | 190 (13.6) | 242 (17.3) | 290 (20.7) | 302 (21.6) | 200 (14.3) | 174 (12.4) |  | 287 (18.4) | 328 (21.0) | 321 (20.5) | 302 (19.3) | 192 (12.3) | 133 (8.5) |  |
| **Age** | 37.96 (1.61) [37.73-38.19] | 42.90 (1.44) [42.71-43.08] | 47.97 (1.35) [47.82-48.13] | 53.04 (1.40) [52.88-53.20] | 58.02 (1.30) [57.84-58.20] | 62.61 (1.25) [62.43-62.80] | <0.001 | 37.80 (1.69) [37.61-38.00] | 42.98 (1.33) [42.84-43.13] | 48.08 (1.41) [47.92-48.23] | 53.03 (1.36) [52.88-53.18] | 57.94 (1.45) [57.73-58.14] | 62.82 (1.37) [62.58-63.05] | <0.001 |
| **BMI** | 25.72 (4.49) [25.08-26.36] | 25.96 (4.38) [25.41-26.52] | 25.71 (3.69) [25.29-26.14] | 25.26 (4.48) [24.75-25.77] | 25.09 (3.79) [24.56-25.62] | 24.46 (3.32) [23.97-24.96] | 0.003 | 25.86 (4.75) [25.30-26.41] | 26.89 (4.97) [26.35-27.43] | 26.91 (4.78) [26.38-27.43] | 28.48 (13.67) [26.93-30.03] | 26.94 (4.68) [26.27-27.60] | 26.03 (4.60) [25.24-26.83] | 0.001 |
| **WC** | 93.94 (10.74) [92.40-95.47] | 95.38 (10.80) [94.00-96.75] | 95.33 (9.95) [94.17-96.49] | 94.75 (11.09) [93.49-96.02] | 95.39 (10.59) [93.90-96.88] | 94.12 (10.55) [92.53-95.71] | 0.564 | 88.78 (10.27) [87.58-89.97] | 91.18 (11.11) [89.97-92.40] | 91.46 (11.22) [90.22-92.69] | 93.54 (11.15) [92.27-94.81] | 92.32 (11.28) [90.71-93.94] | 92.30 (11.37) [90.35-94.26] | <0.001 |
| **SBP** | 122.25 (14.37) [120.18-124.32] | 127.49 (16.38) [125.41-129.57] | 127.01 (17.57) [124.98-129.05] | 129.04 (18.93) [126.89-131.19] | 132.39 (19.34) [129.69-135.08] | 132.62 (17.48) [130.00-135.24] | <0.001 | 117.37 (15.60) [115.56-119.18] | 120.75 (17.73) [118.82-122.69] | 126.08 (19.96) [123.88-128.27] | 129.90 (19.63) [127.66-132.13] | 132.74 (19.94) [129.90-135.58] | 132.98 (20.35) [129.49-136.48] | <0.001 |
| **DBP** | 75.88 (11.40) [74.24-77.52] | 81.34 (11.52) [79.88-82.81] | 80.22 (12.30) [78.79-81.64] | 81.24 (13.06) [79.75-82.72] | 80.65 (11.46) [79.05-82.25] | 80.43 (11.54) [78.70-82.15] | <0.001 | 74.96 (12.32) [73.53-76.39] | 76.63 (11.78) [75.35-77.92] | 79.08 (13.25) [77.62-80.54] | 78.72 (12.03) [77.35-80.09] | 78.87 (10.97) [77.31-80.43] | 78.38 (13.83) [76.00-80.75] | <0.001 |
| **FBG** | 104.37 (37.49) [99.01-109.74] | 111.45 (49.97) [105.12-117.78] | 113.43 (44.42) [108.30-118.57] | 118.66 (53.86) [112.56-124.76] | 120.63 (49.31) [113.75-127.50] | 115.56 (39.75) [109.61-121.51] | 0.008 | 98.37 (27.59) [95.17-101.58] | 105.84 (43.31) [101.14-110.55] | 108.98 (43.96) [104.15-113.81] | 115.46 (52.99) [109.46-121.46] | 109.04 (34.16) [104.18-113.90] | 115.76 (42.92) [108.40-123.12] | <0.001 |
| **Creatinine** | 0.81 (0.13) [0.79-0.83] | 0.81 (0.15) [0.81-0.85] | 0.91 (0.45) [0.83-0.94] | 0.81 (0.22) [0.81-0.86] | 0.81 (0.14) [0.80-0.84] | 0.91 (0.30) [0.83-0.92] | 0.013 | 0.063 (0.24) [0.60-0.66] | 0.061 (0.10) [0.60-0.62] | 0.063 (0.15) [0.61-0.65] | 0.066 (0.35) [0.62-0.70] | 0.063 (0.10) [0.61-0.64] | 0.065 (0.15) [0.62-0.67] | 0.062 |
| **TC** | 188.97 (38.82) [183.42-194.53] | 186.74 (37.86) [181.94-191.53] | 186.62 (38.43) [182.18-191.07] | 181.72 (36.50) [177.58-185.85] | 183.28 (35.72) [178.30-188.26] | 177.47 (36.33) [172.04-182.91] | 0.028 | 173.27 (32.04) [169.55-176.99] | 179.65 (33.80) [175.97-183.32] | 189.93 (35.32) [186.05-193.81] | 198.39 (37.88) [194.10-202.68] | 196.36 (43.62) [190.16-202.57] | 197.68 (55.00) [188.24-207.11] | <0.001 |
| **TG** | 148.72 (130.53) [130.04-167.40] | 163.28 (108.12) [149.59-176.97] | 160.02 (107.38) [147.61-172.43] | 150.65 (96.82) [139.69-161.62] | 147.84 (112.05) [132.22-163.46] | 129.56 (57.47) [120.96-138.16] | 0.024 | 104.73 (49.85) [98.04-110.52] | 113.11 (62.17) [106.13-119.86] | 125.83 (60.55) [119.25-132.48] | 139.45 (88.65) [129.39-149.49] | 135.35 (83.37) [123.35-147.22] | 153.77 (266.04) [108.53-199.40] | <0.001 |
| **L-HDL** | 37.47 (7.31) [36.43-38.52] | 37.77 (7.99) [36.76-38.78] | 37.79 (9.15) [36.73-38.84] | 38.50 (9.61) [37.42-39.59] | 37.45 (8.46) [36.27-38.63] | 38.45 (8.90) [37.12-39.78] | 0.664 | 43.19 (9.54) [42.08-44.29] | 42.82 (8.23) [41.93-43.71] | 43.09 (8.35) [42.17-44.00] | 43.57 (8.90) [42.56-44.58] | 43.04 (8.80) [41.79-44.29] | 43.13 (10.09) [41.40-44.86] | 0.947 |
| **H-LDL** | 133.97 (32.83) [129.28-138.67] | 130.09 (39.61) [125.07-135.10] | 130.86 (35.28) [126.78-134.94] | 125.96 (33.77) [122.14-129.79] | 127.70 (31.66) [123.28-132.11] | 122.53 (33.40) [117.54-127.53] | 0.02 | 118.87 (29.07) [115.49-122.24] | 124.86 (30.72) [121.52-128.20] | 132.60 (36.15) [128.63-136.57] | 137.84 (34.19) [133.97-141.71] | 136.06 (40.97) [130.23-141.89] | 133.65 (34.08) [127.80-139.49] | <0.001 |
| **AST** | 23.57 (10.30) [22.10-25.04] | 23.53 (11.14) [22.11-24.94] | 23.20 (15.16) [21.45-24.95] | 23.10 (13.94) [21.52-24.68] | 21.84 (12.98) [20.03-23.65] | 20.91 (8.67) [19.61-22.20] | 0.217 | 19.17 (6.47) [18.42-19.92] | 18.91 (6.83) [18.16-19.65] | 19.92 (8.68) [18.96-20.87] | 19.60 (6.42) [18.87-20.33] | 19.80 (7.01) [18.80-20.80] | 20.52 (10.00) [18.81-22.24] | 0.264 |
| **ALT** | 25.41 (15.65) [23.17-27.65] | 26.61 (17.30) [24.41-28.80] | 24.95 (16.26) [23.07-26.83] | 21.93 (13.72) [20.38-23.49] | 21.08 (16.83) [18.74-23.43] | 18.82 (10.48) [17.25-20.39] | <0.001 | 15.61 (8.99) [14.56-16.65] | 15.83 (9.79) [14.77-16.89] | 17.65 (11.81) [16.35-18.94] | 17.59 (8.37) [16.65-18.54] | 17.55 (10.49) [16.06-19.04] | 17.94 (10.57) [16.13-19.75] | 0.016 |
| **Uric Acid** | 5.58 (1.35) [5.39-5.77] | 5.62 (1.46) [5.43-5.80] | 5.31 (1.38) [5.16-5.47] | 5.40 (1.37) [5.24-5.55] | 5.18 (1.38) [4.99-5.37] | 5.42 (1.49) [5.20-5.64] | 0.011 | 4.13 (1.27) [3.98-4.28] | 4.20 (1.22) [4.07-4.33] | 4.45 (1.13) [4.33-4.58] | 4.67 (1.23) [4.53-4.81] | 4.68 (1.20) [4.51-4.85] | 4.77 (1.33) [4.54-5.00] | <0.001 |
| **ABI Ratio** | 1.02 (0.06) [1.01-1.03] | 1.03 (0.06) [1.02-1.03] | 1.04 (0.06) [1.03-1.04] | 1.04 (0.06) [1.03-1.04] | 1.03 (0.05) [1.02-1.03] | 1.03 (0.06) [1.02-1.04] | 0.059 | 0.98 (0.07) [0.97-0.98] | 0.99 (0.07) [0.98-0.99] | 1.00 (0.07) [0.98-1.00] | 1.00 (0.07) [0.98-1.00] | 1.00 (1.10) [0.97-1.00] | 1.00 (0.07) [0.98-1.00] | 0.092 |
| **CAC** | 0.23 (0.78) [0.10–0.36] | 0.45 (1.15) [0.31–0.60] | 0.80 (1.56) [0.63–0.98] | 1.41 (2.04) [1.21–1.62] | 2.38 (2.35) [2.12–2.65] | 2.59 (2.36) [2.30–2.89] | <0.001 | 0.10 (0.65) [0.01–0.19] | 0.17 (0.78) [0.07–0.26] | 0.36 (1.07) [0.21–0.50] | 0.96 (1.70) [0.73–1.18] | 1.63 (2.17) [1.32–1.94] | 1.61 (2.15) [1.28–1.93] | <0.001 |
| **cIMT Right** | 0.06 (0.011) [0.055-0.059] | 0.06 (0.012) [0.057-0.059] | 0.06 (0.013) [0.058-0.059] | 0.06 (0.013) [0.061-0.069] | 0.07 (0.055) [0.061-0.069] | 0.07 (0.016) [0.064-0.069] | <0.001 | 0.05 (0.026) [0.051-0.057] | 0.06 (0.038) [0.054-0.062] | 0.06 (0.025) [0.055-0.061] | 0.06 (0.012) [0.058-0.061] | 0.07 (0.068) [0.060-0.080] | 0.07 (0.013) [0.063-0.068] | <0.001 |
| **cIMT Left** | 0.06 (0.028) [0.056-0.064] | 0.06 (0.015) [0.059-0.064] | 0.06 (0.016) [0.060-0.064] | 0.07 (0.017) [0.064-0.074] | 0.07 (0.020) [0.067-0.074] | 0.07 (0.026) [0.070-0.074] | <0.001 | 0.05 (0.10) [0.51-0.054] | 0.06 (0.12) [0.54-0.056] | 0.06 (0.29) [0.58-0.064] | 0.06 (0.14) [0.61-0.065] | 0.07 (0.77) [0.62-0.084] | 0.07 (0.19) [0.66-0.072] | <0.001 |

Values are presented as mean (standard deviation) with 95% confidence intervals in parentheses. The p-values indicate the overall statistical difference across age groups within each sex, calculated using one-way analysis of variance (ANOVA) or the Kruskal–Wallis test, as appropriate. Coronary artery calcium (CAC) scores are reported using log-transformed values [log(CAC + 1)] due to the right-skewed distribution of raw CAC scores. Carotid intima-media thickness (cIMT) values are provided separately for the right and left common carotid arteries. Systolic blood pressure (SBP), diastolic blood pressure (DBP), fasting blood glucose (FBG), total cholesterol (TC), triglycerides (TG), low-density lipoprotein cholesterol (LDL-C), high-density lipoprotein cholesterol (HDL-C), aspartate aminotransferase (AST), alanine aminotransferase (ALT), serum uric acid, serum creatinine, waist circumference (WC), body mass index (BMI), and ankle-brachial index (ABI) were measured using standard protocols. A p-value of less than 0.05 was considered statistically significant.

**Supplementary Table 2:** Clinical and biochemical parameters stratified by coronary artery calcium (CAC) categories and sex

|  | **Men** | | | | | | **Women** | | | | | |
| --- | --- | --- | --- | --- | --- | --- | --- | --- | --- | --- | --- | --- |
| **Parameters/**  **CAC category** | **No CAC (0)** | **Minimal CAC (1-10)** | **Mild CAC (11-100)** | **Moderate CAC (101-400)** | **Severe CAC (>400)** | **p Value** | **No CAC (0)** | **Minimal CAC (1-10)** | **Mild CAC (11-100)** | **Moderate CAC (101-400)** | **Severe CAC (>400)** | **p Value** |
| **Sample n (%)** | 930 (66.5) | 122 (8.7) | 212 (15.2) | 93 (6.7) | 41 (2.9) |  | 1274 (81.5) | 86 (5.5) | 127 (8.1) | 61 (3.9) | 15 (1.0) |  |
| **Age** | 47.85 (7.39)  [47.38-48.33] | 51.36 (7.78) [49.97-52.75] | 54.73 (6.50) [53.85-55.61] | 56.61 (5.70) [55.44-57.79] | 58.17 (4.93) [56.61-59.73] | <0.001 | 47.18 (7.53) [46.76-47.59] | 53.37 (6.83) [51.91-54.84] | 54.63 (5.95) [53.59-55.67] | 55.28 (6.62) [53.58-56.97] | 58.20 (4.30) [55.82-60.58] | <0.001 |
| **BMI** | 25.26 (4.22)  [24.99-25.53] | 26.37 (3.69) [25.71-27.03] | 25.66 (3.97) [25.12-26.20] | 25.16 (3.84) [24.37-25.96] | 25.36 (3.30) [24.32-26.40] | 0.06 | 26.76 (7.95) [26.32-27.20] | 27.98 (4.49) [27.02-28.94] | 27.53 (4.33) [26.77-28.29] | 27.94 (4.35) [26.83-29.06] | 27.47 (5.22) [24.58-30.37] | 0.352 |
| **WC** | 94.41 (10.49) [93.73-95.09] | 96.21 (11.02) [94.22-98.20] | 95.59 (10.76) [94.13-97.06] | 95.96 (11.55) [93.57-98.35] | 95.47 (8.96) [92.60-98.33] | 0.221 | 90.87 (11.12) [90.26-91.49] | 94.09 (9.28) [92.10-96.08] | 93.49 (10.63) [91.63-95.36] | 95.49 (13.13) [92.13-98.85] | 95.07 (9.76) [89.66-100.47] | <0.001 |
| **SBP** | 126.46 (17.31) [125.3-127.58] | 133.84 (17.64) [130.7-137.01] | 130.59 (19.22) [127.9-133.2] | 132.38 (16.76) [128.93-135.83] | 134.46 (16.85) [129.14-139.78] | <0.001 | 123.73 (19.08) [122.68-124.78] | 130.13 (19.26) [126.00-134.26] | 136.00 (19.92) [132.50-139.50] | 133.88 (19.46) [128.86-138.91] | 126.53 (18.13) [116.49-136.57] | <0.001 |
| **DBP** | 79.19 (12.19) [78.41-79.98] | 83.99 (12.59) [81.74-86.25] | 81.46 (11.26) [79.94-82.99] | 80.70 (11.80) [78.27-83.13] | 81.80 (11.31) [78.24-85.37] | <0.001 | 76.91 (12.17) [76.24-77.59] | 79.51 (11.36) [77.08-81.95] | 82.90 (13.42) [80.54-85.25] | 80.10 (13.88) [76.51-83.69] | 75.13 (11.26) [68.90-81.37] | <0.001 |
| **FBG** | 109.34 (42.20) [106.6-112.05] | 121.54 (54.28) [111.8-131.27] | 120.00 (52.96) [112.8-127.2] | 133.09 (59.42) [120.85-145.32] | 132.66 (48.17) [117.45-147.86] | <0.001 | 106.37 (40.32) [104.15-108.59] | 107.70 (40.39) [99.04-116.36] | 117.15 (54.99) [107.49-126.81] | 112.64 (36.56) [103.28-122.00] | 173.80 (68.11) [136.08-211.52] | <0.001 |
| **Creatinine** | 0.84 (0.20)  [0.83-0.86] | 0.85 (0.15) [0.82-5.88] | 0.83 (0.18) [0.81-8.86] | 0.82 (0.12) [0.79-2.84] | 01.02 (01.16) [0.66-1.39] | 0.001 | 0.063 (0.22) [0.062-0.064] | 0.064 (0.12) [0.061-0.067] | 0.064 (0.19) [0.060-0.067] | 0.062 (0.18) [0.058-0.067] | 0.058 (0.09) [0.053-0.063] | 0.882 |
| **TC** | 184.18 (37.33) [181.8-186.58] | 187.98 (39.17) [180.96-195.0] | 187.37 (37.10) [182.3-192.4] | 178.81 (35.99) [171.39-186.22] | 172.10 (37.18) [160.36-183.83] | 0.062 | 184.51 (37.88) [182.43-186.60] | 203.38 (36.19) [195.63-211.14] | 206.48 (45.53) [198.48-214.48] | 195.28 (40.39) [184.94-205.62] | 188.67 (42.95) [164.88-212.45] | <0.001 |
| **TG** | 148.10 (103.73) [141.4-154.78] | 165.29 (107.6) [145.9-184.58] | 159.59 (119.8) [143.3-175.8] | 152.29 (82.86) [135.23-169.35] | 143.63 (85.76) [116.57-170.70] | 0.334 | 122.38 (108.14) [116.44-128.33] | 135.35 (59.10) [122.68-148.02] | 150.25 (88.83) [134.65-165.85] | 116.62 (52.99) [103.05-130.19] | 156.60 (74.00) [115.62-197.58] | 0.025 |
| **L-HDL** | 38.31 (8.77)  [37.75-38.88] | 36.98 (7.04) [35.72-38.24] | 37.88 (9.50) [36.59-39.16] | 36.22 (7.49) [34.67-37.76] | 36.27 (9.19) [33.37-39.17] | 0.079 | 43.36 (9.02) [42.87-43.86] | 42.71 (8.37) [40.92-44.51] | 41.58 (7.47) [40.27-42.89] | 43.20 (8.63) [40.99-45.41] | 39.61 (8.75) [34.76-44.45] | 0.12 |
| **H-LDL** | 128.72 (35.33) [126.4-130.99] | 132.65 (35.98) [126.2-139.10] | 130.41 (33.93) [125.81-135] | 123.78 (30.24) [117.56-130.01] | 115.59 (30.84) [105.85-125.32] | 0.045 | 127.00 (33.12) [125.18-128.82] | 144.57 (33.16) [137.46-151.68] | 145.65 (41.44) [138.38-152.93] | 138.87 (35.64) [129.74-148.00] | 131.07 (37.87) [110.09-152.04] | <0.001 |
| **AST** | 23.18 (13.87) [22.29-24.08] | 22.37 (9.25) [20.72-24.03] | 22.52 (9.11) [21.29-23.76] | 19.94 (7.63) [18.37-21.51] | 23.45 (15.57) [18.53-28.36] | 0.2 | 19.39 (7.05) [19-19.78] | 21.35 (10.05) [21-23.50] | 20.08 (7.14) [20-21.33] | 19.00 (6.27) [19-20.60] | 19.91 (20.12) [19-31.05] | 0.159 |
| **ALT** | 24.10 (16.92) [23.01-25.19] | 22.84 (11.56) [20.77-24.91] | 22.84 (12.50) [21.15-24.53] | 18.81 (9.42) [16.87-20.75] | 20.07 (15.72) [15.11-25.03] | 0.015 | 16.54 (9.81) [16.00-17.08] | 19.04 (11.89) [16.49-21.59] | 18.35 (10.28) [16.55-20.16] | 18.08 (9.61) [15.62-20.54] | 17.33 (14.67) [9.21-25.45] | 0.059 |
| **Uric Acid** | 5.43 (1.37)  [5.34-5.52] | 5.59 (1.55) [5.31-5.87] | 5.38 (1.51) [5.18-5.59] | 5.38 (1.31) [5.11-5.64] | 4.91 (1.36) [4.48-5.34] | 0.113 | 4.35 (1.21) [4.29-4.42] | 4.82 (1.29) [4.55-5.10] | 4.79 (1.34) [4.56-5.03] | 4.87 (1.21) [4.56-5.18] | 4.49 (1.31) [3.76-5.21] | <0.001 |
| **ABI Ratio** | 1.03 (0.06)  [1.03-1.04] | 1.04 (0.06) [1.04-1.05] | 1.03 (0.06) [1.03-1.04] | 1.04 (0.06) [1.04-1.05] | 1.02 (0.06) [1.02-1.04] | 0.475 | 0.99 (0.07) [0.98-0.99] | 0.99 (0.07) [0.97-1.00] | 0.98 (0.07) [0.97-1.00] | 0.97 (0.15) [0.93-1.00] | 0.902 (0.06) [0.98-1.05] | 0.119 |
| **CAC** | 0 | 4.1 (4.2)  [4.05-4.9] | 34.8 (43.5) [39.24-46.09] | 192.2 (115) [195.4-228.5] | 620.8 (551.1) [674.8-1143.3] | <0.001 | 0 | 4.4 (4.8) [4.32-5.62] | 37.6 (40.0) [38.08-46.34] | 170.6 (93.8) [166.76-205.59] | 619.2 (415.6) [518.55-1267.50] | <0.001 |
| **cIMT (cm) Right** | 0.061 (0.028) [0.059-0.062] | 0.061 (0.015) [0.059-0.065] | 0.061 (0.015) [0.059-0.067] | 0.061 (0.013) [0.059-0.067] | 0.071 (0.019) [0.059-0.073] | 0.11 | 0.058 (0.025) [0.056-0.059] | 0.073 (0.068) [0.058-0.087] | 0.062 (0.014) [0.059-0.064] | 0.063 (0.013) [0.060-0.067] | 0.113 (0.207) [0.002-0.227] | <0.001 |
| **cIMT (cm) Left** | 0.063 (0.19)  [0.062-0.064] | 0.066 (0.24) [0.062-0.071] | 0.070 (0.19) [0.067-0.073] | 0.068 (0.22) [0.063-0.072] | 0.077 (0.24) [0.070-0.085] | <0.001 | 0.058 (0.013) [0.057-0.059] | 0.076 (0.078) [0.060-0.093] | 0.065 (0.016) [0.062-0.068] | 0.067 (0.014) [0.064-0.071] | 0.129 (1.229) [0.002-0.256] | <0.001 |

Values are presented as mean (standard deviation) with 95% confidence intervals in square brackets. Coronary artery calcium (CAC) scores were categorized as: No CAC (score = 0), Minimal CAC (1–10), Mild CAC (11–100), Moderate CAC (101–400), and Severe CAC (>400). P-values reflect the overall comparison across CAC categories within each sex, using one-way analysis of variance (ANOVA) or the Kruskal–Wallis test, as appropriate. Coronary artery calcium (CAC) scores are reported as Median (IQR) with 95% confidence intervals. Carotid intima-media thickness (cIMT) values are provided separately for the right and left common carotid arteries. Systolic blood pressure (SBP), diastolic blood pressure (DBP), fasting blood glucose (FBG), total cholesterol (TC), triglycerides (TG), low-density lipoprotein cholesterol (LDL-C), high-density lipoprotein cholesterol (HDL-C), aspartate aminotransferase (AST), alanine aminotransferase (ALT), serum uric acid, serum creatinine, waist circumference (WC), body mass index (BMI), and ankle-brachial index (ABI) were measured using standard protocols. A p-value of less than 0.05 was considered statistically significant.

**Supplementary Table 3:** Age stratified association of metabolic risk factors and CAC.

|  | **Unadjusted** | | **Adjusted*** | |
| --- | --- | --- | --- | --- |
|  | **OR [95% C.I]** | **P Value** | **OR [95% C.I]** | **P Value** |
| **35-45 Years** |  |  |  |  |
| Diabetes | 1.45 [0.84-2.50] | 0.173 | 1.38 [0.80-2.37] | 0.243 |
| Hypertension | 1.85 [1.14-3.01] | 0.012 | 1.62 [0.99-2.64] | 0.054 |
| Dyslipidaemia | 1.27 [0.48-3.32] | 0.626 | 1.18 [0.44-3.14] | 0.734 |
| Obesity | 3.46 [1.70-7.04] | 0.001 | 2.58 [0.60-11.06] | 0.199 |
| Overweight | 2.17 [1.23-3.84] | 0.007 | 1.90 [0.87-4.15] | 0.104 |
| Abnormal IMT | 2.95 [0.87-9.96] | 0.081 | 2.98 [0.85-10.37] | 0.086 |
| Current Smoker | 2.83 [1.60-5.01] | <0.001 | 1.75 [0.94-3.25] | 0.073 |
| Non-HDL | 1.87 [0.93-3.77] | 0.078 | 1.70 [0.83-3.47] | 0.14 |
| **46-55 Years** |  |  |  |  |
| Diabetes | 1.69 [1.26-2.26] | <0.001 | 1.72 [1.27-2.32] | <0.001 |
| Hypertension | 1.75 [1.33-2.29] | <0.001 | 1.65 [1.25-2.18] | <0.001 |
| Dyslipidaemia | 0.97 [0.58-1.60] | 0.909 | 0.97 [0.57-1.62] | 0.907 |
| Obesity | 1.18 [1.77-1.80] | 0.427 | 1.54 [0.85-2.82] | 0.153 |
| Overweight | 1.35 [1.00-1.83] | 0.044 | 1.56 [1.09-2.24] | 0.015 |
| Abnormal IMT | 2.5 [1.39-4.49] | 0.002 | 1.92 [1.05-3.52] | 0.034 |
| Current Smoker | 1.96 [1.40-2.75] | <0.001 | 1.55 [1.06-2.27] | 0.024 |
| Non-HDL | 1.49 [1.02-2.17] | 0.039 | 1.53 [1.06-2.27] | 0.032 |
| **56-65 Years** |  |  |  |  |
| Diabetes | 1.49 [1.07-2.06] | 0.016 | 1.42 [1.02-1.19] | 0.038 |
| Hypertension | 1.221 [0.89-1.67] | 0.213 | 1.18 [0.86-1.63] | 0.298 |
| Dyslipidaemia | 1.35 [0.80-2.26] | 0.253 | 1.38 [0.82-2.33] | 0.222 |
| Obesity | 1.26 [0.73-2.18] | 0.389 | 0.75 [0.26-2.17] | 0.606 |
| Overweight | 1.66 [1.20-2.31] | 0.002 | 1.27 [0.74-2.17] | 0.374 |
| Abnormal IMT | 1.58 [0.96-2.61] | 0.069 | 1.43 [0.86-2.38] | 0.162 |
| Current Smoker | 1.95 [1.29-2.94] | 0.001 | 1.34 [0.85-2.12] | 0.196 |
| Non-HDL | 0.93 [0.61-1.40] | 0.731 | 0.97 [0.64-1.49] | 0.919 |

*Adjusted for Age, Sex, and BMI. Values are represented as OR [95% C.I]. A p-value of less than 0.05 was considered statistically significant.

**Supplementary Table 4: Methodological Comparison of Global Population-Based CAC Studies and the Present REACH-Rural India Study**

|  | **MESA**^1^ | **MASALA^2^** | **CARDIA**^3^ | **DANCAVAS^4^** | **BioImage**^5^ | **SCAPIS**^6^ | **REACH-Rural India (Present Study)** |
| --- | --- | --- | --- | --- | --- | --- | --- |
| **Start** | 2000 | 2010 | 1985 | 2014 | 2008 | 2014 | 2022 |
| **Completion** | 2002 | 2013 | 1986 |  | 2009 | 2018 | 2024 |
| **Country** | USA | USA | USA | Denmark | USA | Sweden | India |
| **Age group (years)** | 45—84 | 40-79 | 18-30 | Only Men age of 65-74 | Men >55-80, Women >60—80 | 50—64 | 35-65 |
| **Sample size** | 6814 (53% women) | 906 (46% women) | 2935 | 45000 | 6102 (56% women) | 30 000 (50% women) | 3006 (52.8% women) |
| **Exclusion criteria Population** | Known CVD, treated cancer | MI, stroke, Ischemic heart attacks, Heart Failure, Cancer, Life expectancy <5 years | Missing CT cases on Year 25 follow up and people who did not appear for follow up | None | Claims of CVD, cancer, etc. | None | MI, stroke, Ischemic heart attacks, Heart Failure, Cancer, Not from rural area, plans to leave the residency in <5 Years |
| **Population Recruitment Type** | Stratified for ethnicity | Simple Random sampling | Stratified for ethnicity and sex | Randomisation using EPIDATA | Members of Humana Health Plan; stratified for ethnicity | Random population Sample | Random convenience sampling |
| **Clinical Parameters** | Anthropometry, Blood pressure, Urine albumin and creatinine, blood collection for further analysis | Anthropometry, Blood pressure, Urine albumin and creatinine, Serum lipid profile, Lipoproteins inflammatory markers, FBG, and 2-hrs GTT | Anthropometry, Blood pressure, Lipid profile, Fasting blood glucose, HbA1C | Anthropometric measurements, Blood pressure, HbA1c, lipid parameters, haemoglobin, creatinine kinase (CK), and alanine aminotransferase (ALAT) | Anthropometric measurements, Ankle brachial Index, Blood glucose, Lipid parameters. | Anthropometric measurements, Blood pressure, HbA1c, Lipid parameters, C-reactive protein, creatinine, ECG, Lung function test | Anthropometric measurements, Ankle brachial Index, ECG, Blood glucose, Lipid parameters, Creatinine. |
| **Imaging** |  |  |  |  |  |  |  |
| **Carotid — ultrasound** | 6814 | 906 | 2935 | NA | 6104 | 30000 | 2920 |
| **Coronary artery calcium** | 6814 | 906 | 2935 | 45000 | 6104 | 30000 | 2961 |
| **Carotid - MRI** | NA | NA | NA | NA | 525 | 3000 | NA |
| **Follow up** | 18 and 36 months | 12 and 24 months | 2,5,7,15,20, & 25 Years | NA | until 600 major atherothrombotic events occur |  | Phone calls after 18 months and hospital visit after 36 months (Results not reported in this manuscript) |

**References:**

1. Bild DE. Multi-Ethnic Study of Atherosclerosis: Objectives and Design. *Am J Epidemiol*. 2002;156(9):871-881. doi:10.1093/aje/kwf113
2. Bhatia HS, Lin F, Thomas IC, et al. Coronary artery calcium incidence and changes using direct plaque measurements: The MASALA study. *Atherosclerosis*. 2022;353:41-46. doi:10.1016/j.atherosclerosis.2022.05.006
3. Carr JJ, Jacobs DR, Terry JG, et al. Association of Coronary Artery Calcium in Adults Aged 32 to 46 Years with Incident Coronary Heart Disease and Death. JAMA Cardiol. 2017;2(4):391. doi:10.1001/jamacardio.2016.5493**.**
4. Lindholt JS, Søgaard R, Rasmussen LM, et al. Five-Year Outcomes of the Danish Cardiovascular Screening (DANCAVAS) Trial. *New England Journal of Medicine*. 2022;387(15):1385-1394. doi:10.1056/NEJMoa2208681
5. Baber U, Mehran R, Sartori S, Schoos MM, Sillesen H, Muntendam P, Garcia MJ, Gregson J, Pocock S, Falk E, Fuster V. Prevalence, impact, and predictive value of detecting subclinical coronary and carotid atherosclerosis in asymptomatic adults: the BioImage study. J Am Coll Cardiol. 2015 Mar 24;65(11):1065-74.
6. Bergström G, Persson M, Adiels M, et al. Prevalence of Subclinical Coronary Artery Atherosclerosis in the General Population. Circulation. 2021;144(12):916-929. doi:10.1161/CIRCULATIONAHA.121.055340
